# Immunological Mechanisms in Allergic Diseases and Allergen Tolerance: The Role of Treg Cells

**DOI:** 10.1155/2018/6012053

**Published:** 2018-06-14

**Authors:** D. Calzada, S. Baos, L. Cremades-Jimeno, B. Cárdaba

**Affiliations:** ^1^Immunology Department, IIS-Fundación Jiménez Díaz, UAM, Madrid, Spain; ^2^CIBERES, CIBER of Respiratory Diseases, Madrid, Spain

## Abstract

The immune system regulates itself to establish an appropriate immune response to potentially harmful pathogens while tolerating harmless environmental antigens and self-antigens. A central role in this balance is played by regulatory T cells (Tregs) through various ways of actions. By means of molecule secretion and cell-cell contact mechanisms, Tregs may have the capacity to modulate effector T cells and suppress the action of proinflammatory cytokines across a broad range of cell types. As a result, abnormal regulatory T cell function has been pointed as a main cause in the development of allergic diseases, a major public health problem in industrialized countries, with a high socioeconomic impact. This prevalence and impact have created an international interest in improving the allergy diagnosis and therapy. Additionally, research has sought to gain a better understanding of the molecular mechanisms underlining this kind of disease, in order to a better management. At this respect, the role of Treg cells is one of the most promising areas of research, mainly because of their potential use as new immunotherapeutical approaches. Therefore, the aim of this review is to update the existing knowledge of the role of Tregs in this pathology deepening in their implication in allergen-specific therapy (AIT).

## 1. Introduction: Current Knowledge about Treg Cells

The immune system (IS) requires tight control to protect the organism from exaggerated stimulatory signals triggered by harmless antigens, such as self-antigens and environmental substances. Depending on the nature of the antigen, an imbalance in the regulatory mechanisms of the IS can lead to autoimmune disorders or allergic diseases in genetically predisposed subjects [1, 2].

The induction of a tolerant state in peripheral T cells represents a key step in healthy immune responses to antigens. The first hypothesis to explain the break of this tolerance was based on the dichotomy between Th1 and Th2 lymphocytes. Years later, the hygiene hypothesis suggested that the lack of early childhood exposure to infectious agents and parasites could increase the risk of the susceptibility to suppress the correct development of the IS [3, 4].

Currently, there are several evidences that peripheral T cell tolerance is involved in the regulation of the IS. This regulation is characterized by functional inactivation of the cells in contact with the antigen, which in turn eliminates both the proliferative response and cytokine secretion. Several T cell subtypes with immunosuppressive function have been widely studied, and these are generically named regulatory T cells (Tregs) [5, 6]. Tregs suppress inflammation by upregulating immunosuppressive molecules and inhibiting the cells' tissue homing.

Numerous studies have identified Tregs as important immunoregulators in many inflammatory and autoimmune conditions including asthma, multiple esclerosis, and type I diabetes [7]. Additionally, Tregs are phenotypic and functionally specialized according to tissue localization, disease state, activation, and differentiation status [8–11] and are able to play different roles in disease and health [12, 13]. For these reasons, Tregs have been extensively studied and treated as a promising potential therapeutic tool in different types of diseases [14, 15].

Though this review focuses on the function of the Tregs, it is important to keep in mind that these cells do not work in isolation. In fact, there exists a complex regulatory T cell system, which includes several populations of immunosuppressive cells as myeloid-derived suppressor cells (MDSCs), regulatory B cells (Bregs), regulatory *γδ* T cells (*γδ*-*Τ*regs), and immunosuppressive plasmocytes (ISPC), a regulatory subset of ILCs (innate lymphoid cells) and so on with connected functions that work together [14].

### 1.1. Treg Cell Types

Different types of Tregs have been described [6]. These can be classified into two main categories according to their origin: natural Tregs with thymic origin, which mediate tolerance to self-antigens, and peripheral or induced Tregs, which are derived from a pool of naïve conventional CD4^+^ T cells after exposure to antigens and in the presence of TGF-*β* [16] and regulate the response to nonself antigens [17]. Both of these Treg subsets play a key function in the maintenance of peripheral tolerance, but due to the nonoverlapping T cell receptor (TCR) repertoires, their actions are directed at different antigens. Although currently, there are no exclusive markers for Tregs, both types express FOXP3, a member of the forkhead or winged helix family of transcriptor factors. Indeed, FOXP3 was proposed as a master switch for Treg development and function in mice and humans [18–21]. FOXP3 controls several cell lineages and develops the differentiation of CD4^+^CD25^+^ FOXP3^+^Tregs, the most physiologically significant subtype of these cells [22].

Although the most widely studied regulatory T cells are those with FOXP3, there are also populations of Tregs that do not express FOXP3. These include three main kinds of T cells: Tr1 cells, a population activated in the periphery after antigenic stimulation in the presence of IL-10 and which express the surface markers LAG-3 (lymphocyte-activation gene 3) and CD49b in the face of absent FOXP3 and CD25 expression; Th3 cells, which are also differentiated in the periphery and these Tregs mediate the cell suppression by secreting the cytokine TGF-*β*; and finally, CD8^+^ Tregs [23], described as antigen-specific memory T cells with Treg properties, which may regulate immune responsiveness by production of IL-10, TGF-*β*1, and IFN*γ* though the exact mechanisms underlying this suppression are still largely unknown.

### 1.2. The Central Role of FOXP3

The relevance of FOXP3 in humans was recognized after the discovery of its implication in X-linked immune dysregulation, polyendocrinopathy syndrome (IPEX). IPEX is characterized by a high incidence of autoimmune and allergic diseases, including early-onset diabetes mellitus and other endocrinopathies, enteropathies, and diseases caused by severe allergic inflammation such as eczema, food allergies, and eosinophil-mediated inflammation [22, 24]. Patients with this pathology present mutations in FOXP3 with low levels of circulating Tregs. Indeed, it has been demonstrated that some single nucleotide polymorphisms (SNPs) in the *FOXP3* gene are associated with higher susceptibility to develop allergies [25, 26] and other immune diseases [27, 28]. Given the importance of FOXP3 in the control of the immune response, the factors which in turn control *FOXP3* expression have become a topic of interest. In fact, peripheral Tregs are known to be less stable than nTregs and under different inflammatory conditions can lose FOXP3 expression (ex-Treg) and adopt various T-helper-cell-like phenotypes. Epigenetic modifications, which can target histones (by acetylation) or DNA directly (by methylation in CpG motifs in noncoding regions in the *FOXP3* locus) could regulate the gene expression profile [24, 29]. One explanation for the lack of stability of pTregs was the methylation status of the conserved noncoding region 2 (CNS2) of the *Foxp3* gene. This locus, which acts to maintain Treg lineage identity under inflammatory conditions, is known to be stably hypomethylated in nTreg whereas it is incompletely demethylated in pTregs [30].

In addition to the transcriptional control of the *Foxp3* gene, the stability of FOXP3 expression is also determined at the posttranscriptional level. For example, Tregs respond to stress signals elicited by proinflammatory cytokines and lipopolysaccharides by degrading FOXP3 protein to then acquiring a T-effector-cell-like phenotype [31–33].

### 1.3. Mechanisms Involved in the Modulation of FOXP3 and Treg Cells

In view of the clinical relevance of this gene, elucidating the main mechanisms involved in the regulation of FOXP3 expression and Treg function could be very useful in the effort to control immune-dysregulated disorders and in understanding the physiological role in health and disease of this Treg instability. Thus, an increasing number of studies have been published in recent years to describe the different molecular mechanisms related to FOXP3 instability, in order to determine the expression of transcription factors and receptors that enable the suppressive functions of Tregs to operate in inflammatory and noninflammatory conditions. Examples of such researches include the description in Tregs of a positive feedback between USP21 (an E3 deubiquitinase), GATA3 (an essential and sufficient transcription factor for the polarization and function of the Th2 cell lineage), and FOXP3, to promote FOXP3 expression and thus modulate Treg activity [34]. This last report proposed that after TCR stimulation, FOXP3 upregulates USP21 transcription. USP21 could act as a GATA3 deubiquitinase and to stabilize GATA3. This mechanism may contribute to the upregulation of GATA3 expression in Tregs and can be strengthened in a FOXP3-dependent matter. The study also highlighted the possibility that GATA3 could recruit USP21 to the FOXP3 complex to prevent FOXP3 degradation. This work proposes that in Tregs, USP21, GATA3, and FOXP3 may form a positive loop to promote FOXP3 expression and thus modulate Treg activity. Years later [35], it was described how USP21 stabilizes the FOXP3 protein by mediating its deubiquitination and controls Treg lineage stability *in vivo*.

Another regulator proposed was SOCS2, a suppressor of cytokine signaling (SOCS) proteins, which inhibits the development of Th2 cells and allergic immune responses. It was described as being highly expressed in pTregs and a requisite for the stable expression of Foxp3 in these Tregs, *in vitr*o and *in vivo*, but with no effect on nTreg development or function. Interestingly, pTreg stability was induced by SOCS2 downregulating IL-4 signaling [36].

Recently, MALT1, a nuclear factor-*κ*B signaling mediator mucosa-associated lymphoid tissue lymphoma translocation protein 1, has been described as an important novel regulator of nTregs and pTregs [37]. The report describes a dual role of MALT1, citing it as crucial for nTreg development and thus important for central tolerance and also in the periphery, where MALT1 determines the threshold for the differentiation of naïve T cells into functional pTregs. Although MALT1 had no influence on the suppressive function of pTregs, it was seen to be able to limit further induction of pTregs at sites of inflammation by downregulating Toll-like-receptor (TLR) 2 expression. In this respect, the influence of TLR signaling on Tregs is quite controversial but it is generally accepted that TLR2, 4, or 5 engagements can enhance Treg cell function, survival, and/or proliferation [38–41].

Interestingly, it has been demonstrated that Helios (a member of the ikaros family of transcription factors) expression by Tregs is key to supporting their suppressive functions and phenotypic stability during inflammation [42].

On the other hand, Treg functions can also be regulated by endogenous danger signals, or alarmins, which are released by epithelial cells at the mucosal barrier. One such alarmin is interleukin- (IL-) 33, a cytokine that is released from epithelial and endothelial cells at barrier surfaces upon tissue stress or damage and that was primarily implicated in the induction of Th2-type immune responses. More recently [43], however, their pleitropic role has been demonstrated as this cytokine is able to mediate immunosuppression and tissue repair by activating Tregs and promoting M2 macrophage polarization.

Finally, another important modulator of tolerance is the microbiota. It has been suggested that the microbiota can promote tolerant or proinflammatory cell subtypes. This aspect has been extensively studied in the context of allergic diseases [44, 45]. The release of microbiotal products can interact directly with immune cells and their innate receptors. Therefore, proallergic or protolerant bacterial species can affect the development of Tregs and Th2 subtypes, thereby increasing the production of IL-10 or IL-4 and IL-13, respectively.

### 1.4. General Mechanisms of Treg Action

The main role of all Treg subsets is to maintain the integrity of the organism by avoiding excessive immune responses thereby preserving a state of tolerance to innocuous substances through the secretion of soluble factors and by direct contact (cell-to-cell). A variety of molecules has been found to be involved in Treg-mediated suppressive activities. The main mechanisms of Treg action include regulatory cytokine production such as IL-10, IL-35, and TGF-*β*; the metabolic disruption mechanisms: CD25, cAMP, histamin receptor 2, adenosine receptor 2, CD39, and CD73; mechanisms with targets in DCs such as cytotoxic T lymphocyte antigen-4 (CTLA-4), program death-1 (PD-1), and cytolysis mechanism (granzymes A and B) [14, 46, 47].

As detailed above, it has been described how the suppressive functions of Tregs are induced under inflammatory conditions by the specific expression of receptors and transcription factors. By way of example, GATA3 expression by Tregs is triggered by TCR activation and is required to maintain FOXP3 expression and allow accumulation of Tregs at inflamed sites [48]. GATA3 expression in Tregs appears to be essential in limiting Tregs producing effector cytokines within inflamed tissues [17].

Recently, it has also been described that Tregs secrete microRNAs which could be implicated in inhibiting T effector cells, thus opening a new area of interest in Treg-mediated suppressive mechanisms [23].

**Figure 1:**
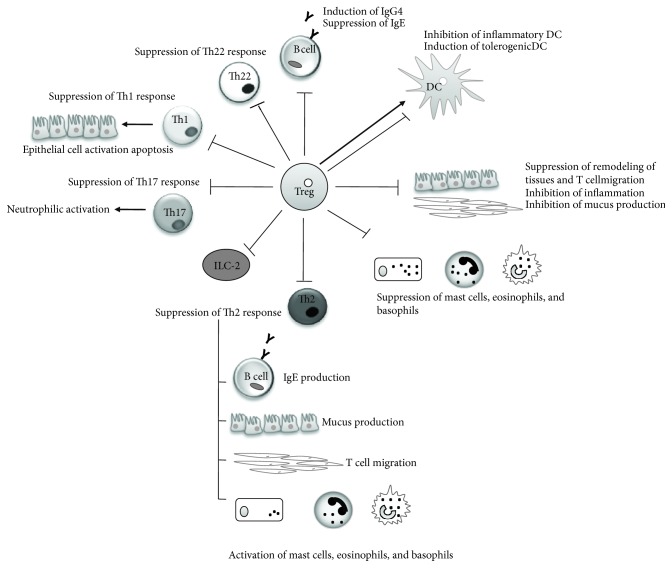
Main roles proposed to Tregs. Regulatory T cells could mediate the healthy immune response through different modes of action. They are able to suppress the inflammatory dendritic cells (DCs), inhibit the activation of effector T cells (Th1, Th2, Th22, and Th17) and type 2 innate lymphoid cells (ILC-2), block the secretion of inflammatory antibodies by antigen-specific B cells, and inhibit the activation of basophils, mast cells, and eosinophils.

To summarize, although the complex functioning of the regulatory network is not well known, Tregs are able to maintain tolerance by multiple mechanisms.  Figure 1 summarizes the main roles proposed for Tregs. These cells could act at the initiation of adequate specific antigenic immune response, promoting tolerogenic DC phenotypes, inhibiting the inflammatory ones [49]. It has also been demonstrated that the correct capacity of DCs to induce a tolerogenic response depends on the particular subsets, maturation stages, and several exogenous signals such as microbiota, histamine, adenosine, flavonoids, vitamin D3 metabolites, or retinoic acid [46]. Suppression of DCs appears to be mediated through CTLA-4 (constitutively expressed in Tregs, as a negative costimulatory molecule which is essential to their suppressive functions), LAG-3 (lymphocyte-activation gene 3), and LFA-1 (leukocyte function-associated antigen-1). Tregs also act directly on DCs by decreasing the surface expression of CD80/CD86 and blocking the allergen-specific Th2 cell immune response. In addition, Tregs could inhibit Th development: Tregs suppress the activation process of Th2 cells, reducing the secretion of inflammatory cytokines such as IL-4, IL-5, IL-9, and IL-13 and in Th1 cells, by inhibiting INF-*γ* secretion, Th17 cell response (IL-17-secreting cells) and Th22, which predominantly produce IL-22 [23]. It has been demonstrated that peripherally induced Tregs suppress the group 2 innate lymphoid (ILC-2) response and its inflammatory cytokines, IL-5 and IL-13 [23]. In addition, Tregs may control effector cells by inhibiting the maturation and degranulation of basophils and mast cells, thus reducing the expression of Fc*ε*RI and the degranulation via OX40-OX40L interactions. Tregs also prevent the infiltration of eosinophils and T cells into damaged tissue. Also, Tregs could interact with resident tissue cells by preventing damage and contributing to tissue remodeling [46]. For this reason, Tregs are involved in the reduction of local inflammation and contribute to the repair of damaged tissue [50–52].

Tregs also could modulate the humoral immune response. These cells may directly inhibit the progress of antigen-specific B cells, reducing the production of immunoglobulin (Ig) type E (IgE) and increasing the levels of anti-inflammatory immunoglobulin such as IgG4 [53]. This is another point justifying Tregs as excellent candidates for regulating allergic diseases.

## 2. Allergic Response: An Imbalance of Regulatory Mechanisms

Regulation of IS a general process that allows inflammation to be attenuated. Defective immunosuppressive mechanisms by Tregs could explain the development of allergic reactions. Allergic diseases are highly complex adverse reactions of the IS against various innocuous substances. Although the population is continuously exposed to a wide range of allergens, not everyone develops this kind of disease. The reasons why some individuals suffer from allergic diseases while others do not are far from clear. The pathophysiology of allergic diseases is complex and may be influenced by many factors, including genetic susceptibility as well as aspects of the microenvironment, such as allergen dose and route of exposure. In this sense, clinical manifestations depend on the nature of the allergen and the part of the organism affected. The most common symptoms of allergic diseases include allergic rhinoconjunctivitis, allergic asthma, atopic dermatitis, food allergy, and anaphylaxis [54].

**Figure 2:**
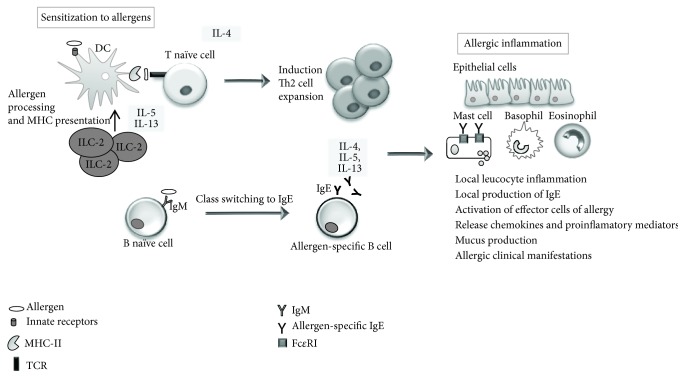
Mechanisms involved in allergic reactions. Sensitization to a specific antigen is a prerequisite for the onset of allergic diseases. Differentiation and expansion to Th2 cell subtypes lead to the production of inflammatory cytokines (IL-4, IL-5, and IL-13). They drive immunoglobulin E (IgE) class-switch in B cells and the recruitment and activation of proinflammatory cells (i.e., eosinophils and mast cells) in mucosal target organs. These activations contribute to the development of the inflammation and the symptoms of allergic disease.

In the allergic response, the IS must recognize the pathogenic stimuli and induce a vigorous immune response. Sensitization to a specific antigen is a prerequisite: specific regions of antigens called epitopes are recognized by naïve T and B lymphocytes. First, the allergens are recognized and presented to naïve T cells by specific major histocompatibility complex (MHC) class II antigens expressed on the surface of antigen-presenting cells (APC). T cell activation induces the differentiation and expansion of T helper type 2 (Th2) cells. The key cytokines responsible for the allergic response include interleukin- (IL-) 4, IL-5, and IL-13, as well as innate lymphoid (ILC-2) cells which may amplify and maintain local Th2-driven allergic inflammation by secreting Th2 cytokines, particularly IL-5 and IL-13 [55]. These ILs act on B cells, promoting immunoglobulin (Ig) class switching to Ig type E (IgE). Allergen-specific IgE antibodies bind to high-affinity receptors for IgE (Fc*ε*RI) expressed on mast cells and basophils. Repeated exposure to the allergen causes the cross-linking of Fc*ε*RI-bound IgE, stimulating the release of histamine and other mediators responsible for the immediate symptoms of allergic disease. The late phase of an allergic reaction occurs 6–12 hours after allergen exposure, when allergen-specific cells are reactivated and expanded locally. Effector cells (i.e., mast cells, basophils, and specifically, eosinophils) release additional inflammatory mediators and cytokines, perpetuating the proinflammatory response. This phase is responsible for the symptoms of allergic diseases, and continuous exposure to the allergen causes disease chronicity (Figure 2) [1, 56].

Currently, the prevalence of allergic diseases has increased substantially, with allergies affecting up to 25% of the population in industrialized societies and constitute a major public health problem with a high socioeconomic impact [46]. Although allergic symptoms can often be suppressed using anti-inflammatory drugs, allergen-specific immunotherapy (AIT) remains as the only treatment that can cure allergic diseases. AIT has been applied since 1911 and was reported first by De Martinis et al. [57]. Their assays proved that subcutaneous injection of grass pollen extract modulated the course of the disease, and more importantly, these effects remained for more than a year after the conclusion of treatment. The method used by the two researchers was accepted by the scientific community, leading to an increase in this type of studies in subsequent years. Nowadays, AIT is a routine part of clinical practice in allergy [57].

## 3. Induction of Peripheral Tolerance in Allergy by AIT

AIT is considered a therapeutic vaccine because it uses the patient IS to establish tolerance against specific antigens, providing clinical efficacy with a long-term benefit. However, although the molecular mechanisms involved in the AIT response have been extensively studied, they have not been elucidated in their entirety [58–60].

**Figure 3:**
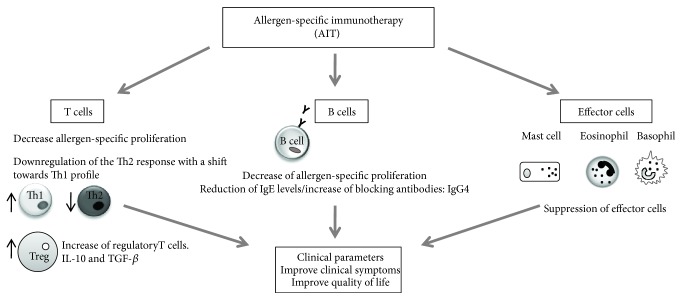
Effects of AIT. AIT induces a state of tolerance based on the increase of Treg functions and the inhibition of the Th2 cell response. The production of blocking antibodies and the suppression of mast cells, basophils, and eosinophils also contribute to the restoration of the appropriate immune response to allergens, by ameliorating the chronic inflammation that occurs in allergic diseases.

AIT is believed to promote the absence of proinflammatory signals influenced by the maturation of dendritic cells (DC) into a tolerogenic response [61], thus inducing major changes in allergen-specific T cell subsets: it leads to a downregulation of the Th2 response with a shift towards the Th1 profile. Allergen-specific T cells with a regulatory phenotype are also promoted, and their presence is associated with an increase in the suppressive cytokines such as IL-10 and TGF-*β* [53, 62–64]. Through this fact, AIT plays an essential role in changing antibody isotypes. Many studies have shown a decrease of allergen-specific IgE antibodies (inflammatory response) in serum associated with high levels of the allergen-specific IgG4 antibody, a noninflammatory or protective immunoglobulin, in the context of allergic response [44]. IgG4 secreted by regulatory B cells [65] could act as a blocking antibody in competition with the antigen-binding IgE present on the surface of mast cells and basophils, which would limit activation and degranulation [66]. AIT and this promoting of Tregs also suppress allergic inflammation induced by mast cells, basophils, and eosinophils. The recruitment of these cells to the site of allergen exposure and their ability to release mediators are reduced in treated patients, decreasing the inflammatory response of tissues [63]. Due to these capacities, AIT reduces the allergic symptoms in a significant fraction of treated individuals and improves their quality of life (Figure 3) [54].

## 4. Regulatory Cytokines in Allergen Tolerance and Specific Immunotherapy

One of the most important mechanisms of Tregs is the secretion of some soluble mediators, such as suppressive cytokines (IL-10, IL-35, and TGF-*β*). Their importance in allergen tolerance and their modulation with AIT have been widely studied.

### 4.1. Cytokine 10 (IL-10)

This cytokine is formed by two subunits of 178 amino acids [67, 68]. It is synthesized and secreted by a wide range of cell types, including Th cells, monocytes, macrophages, and dendritic cells [69, 70].

Its receptor is formed by two chains: IL10-R1, expressed only in target cells (T, B, NK cells, monocytes, mast cells, and dendritic cells) and IL10-R2, expressed ubiquitously. The junction ligand-receptor produces signaling by phosphorylation of some proteins that induce the activation of signal transducer and activator of transcription 3 (STAT3), which induce the expression of suppressor of cytokine signaling 3 (SOCS3) and several preapoptotic genes, as well as the inhibition in the production of proinflammatory cytokines such as TNF-*α*.

Due to these activations, IL-10 has broad immunosuppressive and anti-inflammatory capacities. It is a potent inhibitor of proinflammatory cytokine production and their receptors. It inhibits several molecules involved in the antigen presentation to dampen TCD4^+^ cell activation.

IL-10 produced by Tregs plays an essential role in protecting the host from exaggerated inflammatory responses to pathogens as well as autoimmune diseases. In addition, its implication in tolerogenic and allergic responses has been extensively studied and the protective role it plays nowadays is well-established. It has been demonstrated that dendritic cells from the respiratory system of healthy controls express higher levels of IL-10 than allergic patients (with rhinitis and asthma) [71, 72]. IL-10 modulates the activity of numerous cells involved in allergic diseases, mast cells, Th2 cells, and eosinophils. Several researchers have shown that Treg-specific deletion of IL-10 promoted allergic inflammation [73] suggesting that Treg-derived IL-10 plays a “privileged,” nonredundant role in the induction of immune tolerance in allergic airway inflammation. High levels of allergens, as cat allergens, induce IL-10 production by Tr1 cells associated with the secretion of IgG4 and amelioration of clinical symptoms [74]. Also, the exposure to high doses of bee venom in beekeepers has demonstrated a natural mechanism of immune tolerance owing to the expansion of IL-10 secreting Tr1 cells [75]. Furthermore, numerous clinical trials have reported that specific treatments for allergies increase IL-10 levels [76–78].

### 4.2. Tumor Growth Factor-*β* (TGF-*β*)

TGF-*β* is a member of a complex superfamily, being TGF-*β*1 the most widely studied member [79, 80].

TGF-*β* is a pleiotropic cytokine required for the maintenance of peripheral tolerance. TGF-*β* regulates lymphocyte homeostasis by inhibiting Th2 and Th1 cell responses as well as the conversion of *naïve* T cells into peripheral regulatory T cells by the stimulation of FOXP3 expression in a paracrine feedback loop, which promotes the generation of CD4^+^ CD25^+^ Tregs able to inhibit allergic airway disease [81–83].

TGF-*β* also inhibits macrophage proliferation and function, inhibits the secretion of antibodies by B cells, and also blocks the expression of Fc*ε*RI in mast cells. In damaged tissues, TGF-*β* regulates airway inflammatory response, inducing fibrosis [84].

### 4.3. Cytokine 35 (IL-35)

Il-35 is a newly discovered member of the IL-12 family. It is a heterodimer composed of a subunit of IL-12 (p35, IL-12*α*) and the Epstein-Barr virus-induced gene 3 (EBI3) [85]. While the other IL-12 family members (IL-13, IL-23, and IL-27) are considered proinflammatory cytokines, IL-35 is secreted by Tregs and was identified as an anti-inflammatory and suppressive cytokine [8, 22] in an inducible manner [86].

Tregs deficient in Ebi3 or IL-12p35 are functionally defective *in vitro* and *in vivo* [17]. Several findings lend support to the importance of these Tregs in some respiratory diseases. Recent findings suggest that expression of IL-35 is abnormal in asthma and plays an important role in the pathogenesis of allergy diseases [87]. It has also been demonstrated how people with asthma and chronic obstructive pulmonary disease (COPD) have low levels of this cytokine and after sublingual immunotherapy, IL35 serum levels increased, being this increase associated with an improvement in clinical symptoms.

## 5. MicroRNAs as a New Mechanism of Suppression by Tregs

MicroRNAs are short noncoding RNA molecules which have emerged as important regulators of immune response. MicroRNAs are fundamental in Treg development and function. miR-155, miR-15b/16, miR-24, and miR-29a were described as important players in Treg differentiation and maintenance [23, 88].

Furthermore, this mechanism for cell communication mediated by exosomes can inhibit the action of T effector cells. Specifically, Tregs released Let7d to Th1 cells regulating Th1 response [89] and miR-21 in the control of Th2 inflammation. Therefore, Tregs are able to capture and deliver different miRNAs and other substances to different cells at different times, depending on the specific situation, to control Treg function and development status [90].

## 6. Conclusions and Future Perspective

The immune system requires correct functioning and fine balance that it are controlled by the development and maintenance of a complex network of regulatory mechanisms, where regulatory cells play essential roles. By gaining a fuller understanding of the heterogeneity of Treg populations and the appropriate suppressive function system in inflammatory conditions, we may be able to devise novel therapeutic approaches to inhibit this kind of disease. For this reason, we have reviewed the general mechanisms of Tregs and the immunologic mechanisms involved in allergy and allergen tolerance, where Tregs could act as the nucleus in enforcing *healthy* immune responses to allergens. Tregs are capable of suppressing conventional T cells, APCs, and B cells by molecule secretion and cell-cell contact mechanisms.

Recovery of the correct immune tolerance response in inflammatory diseases such as allergy is an attractive target for immunotherapy, and Tregs could play a main role in this pursuit as new therapy tools. However, one important aspect that should be studied in depth is Th reprogramming of Tregs in allergic diseases. Nowadays, a dynamic view of Tregs is emerging in allergic diseases by which Tregs are seen as playing a central and determining role, not only in tolerance induction but also, when destabilized and reprogrammed, in mediating disease pathogenesis, severity, and chronicity [44]. New findings on the pathways and mechanisms implicated in this regard could provide unique tools to control Treg function to treat allergic diseases.
